# Mechanical communication within the microtubule through network-based analysis of tubulin dynamics

**DOI:** 10.1007/s10237-023-01792-5

**Published:** 2023-12-07

**Authors:** Marco Cannariato, Eric A. Zizzi, Lorenzo Pallante, Marcello Miceli, Marco A. Deriu

**Affiliations:** PolitoBIOMed Lab, Department of Mechanical and Aerospace Engineering, Politecnico di Torino, Turin, Italy

**Keywords:** Microtubule, Molecular dynamics, Network analysis, Structural communication

## Abstract

The identification of the mechanisms underlying the transfer of mechanical vibrations in protein complexes is crucial to understand how these super-assemblies are stabilized to perform specific functions within the cell. In this context, the study of the structural communication and the propagation of mechanical stimuli within the microtubule (MT) is important given the pivotal role of the latter in cell viability. In this study, we employed molecular modelling and the dynamical network analysis approaches to analyse the MT. The results highlight that $\beta$-tubulin drives the transfer of mechanical information between protofilaments (PFs), which is altered at the seam due to a different interaction pattern. Moreover, while the key residues involved in the structural communication along the PF are generally conserved, a higher diversity was observed for amino acids mediating the lateral communication. Taken together, these results might explain why MTs with different PF numbers are formed in different organisms or with different $\beta$-tubulin isotypes.

**Supplementary Information:**

The online version contains supplementary material available at 10.1007/s10237-023-01792-5.

## Introduction

Microtubules (MTs) are long filaments shaped as hollow cylinders composing the cell cytoskeleton, together with actin microfilaments and intermediate filaments. They provide structural support to the cell and play a pivotal role in many processes such as vesicular trafficking (Ishikawa 2012; Guedes-Dias and Holzbaur 2019), cell motility (Nachury and Mick 2019) and chromosome segregation during mitosis (Prosser and Pelletier 2017; Vicente and Wordeman 2019). MTs are composed of laterally coupled protofilaments (PFs), which are in turn formed by $\alpha$/$\beta$-tubulin heterodimers bound together in a head-to-tail fashion. Following the nomenclature established in previous literature, the structure of *α* and *β* tubulin is similar and composed by three main domains (Nogales et al. 1998): (i) the N-terminal (N) domain faces the growing MT end and can bind GTP, (ii) the intermediate (I) domain located at the shrinking MT end, and (iii) the C-terminal (C) domain exposed towards the outside of the MT wall. While the α tubulin GTP-binding site is non-exchangeable and always occupied by GTP, the one in *β* tubulin is exchangeable and catalyzes the hydrolysis of GTP to GDP when the heterodimer is assembled into the MT lattice. Adjacent PFs are vertically shifted with respect to each other, meaning that circumferentially bound tubulins form a helix; the helix rise may change with the number of PFs composing the MT, which can range from 9 to 16 depending on the organism and the cell type, with the most common lattice being formed by 13 PF (Chaaban and Brouhard 2017). The influence of cell type on the MT architecture, in particular, has led to the hypothesis that the cell might control the number of PFs to tune MT properties, such as its mechanics (Chaaban and Brouhard 2017; Ferreira et al. 2023). In this context, previous works showed that the MT architecture is related to the tubulin isotype expression (Raff et al. 1997; Fukushige et al. 1999), which is specific to cell types (Leandro-García et al. 2010). In particular, it was observed that MTs composed of different $\beta$-tubulin isotypes in human cells are characterized by different numbers of PFs (Ti et al. 2018).

**Fig. 1:**
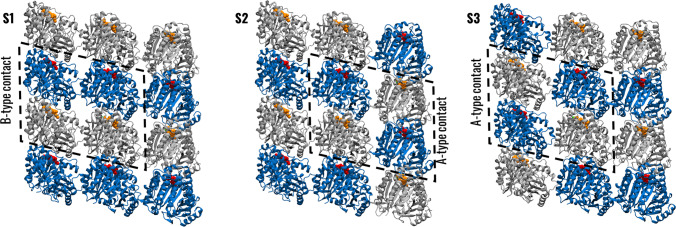
Simulated systems. Visual rendering of the simulated sheets highlighting the B-type and A-type contacts. The view is from outside the MT. $\beta$-tubulin is represented in blue, $\alpha$-tubulin in grey, GTP in orange, and GDP in red

Within the MT lattice, tubulins of adjacent PFs form B-type contacts, which are defined as *α*–*α* or *β*–*β* tubulin contacts or, more in general, homophilic interactions between laterally coupled tubulins. However, depending on the vertical shift, in several architectures, such as MTs with PF numbers ranging from 11 to 14, an irregularity originates so that adjacent tubulins form instead heterophilic *α*-*β* interactions, in the so-called A-type contacts (see Fig. 1); this region is known as the seam. The described architecture, characterized mainly by B-type joins, has been defined as B-lattice. The existence of an A-lattice, formed exclusively by A-type contacts, has been hypothesized, but there are no confirmed examples of such an architecture (Howard 2001). Previous studies have shown that the B-type contact is energetically favoured with respect to the A-type join, and that lateral interactions are generally weaker than longitudinal ones (Sept et al. 2009; Tong and Voth 2020), making the seam the intrinsically weaker region of the B-lattice. Hence, the seam is usually identified as a region where the MT depolymerization starts due to the sole thermal fluctuations. However, recent work showed no substantial difference in the mechanical and energetical stability of the seam with respect to the rest of the architecture (Szatkowski et al. 2019). Moreover, although an extensive computational analysis of a MT showed how PFs have a higher tendency of separating at the seam compared to the rest of the structure, depolymerization is still statistically more favourable to initiate at a B-type interface due to their higher number (Igaev and Grubmüller 2022).

In this context, computational molecular modelling techniques such as molecular dynamics (MD) simulations have been broadly employed to characterize protein dynamics and mechanics, also in the field of MTs (Soncini et al. 2007; Apicella et al. 2013; Craddock et al. 2014; Li et al. 2019; Zizzi et al. 2022). Moreover, the analysis of MD simulations with network approaches has emerged as a powerful methodology to study complex phenomena including allosterism, intra-protein, and inter-protein structural communication, but also to identify crucial residues for the functions and mechanical stability of proteins (Melo et al. 2020; Manrique et al. 2023). In particular, the study of the structural and mechanical communication within the protein complexes such as MTs is of particular interest as it may give detailed insights into the mechanical stability of such systems. It is worth highlighting that in this work the term, mechanical communication refers to the way and the possible pathways through which mechanical and vibrational stimuli could propagate inside the MT lattice. Within this scope, our study aims to couple MD and network analysis to characterize the mechanical communication within a GDP-bound MT also in the light of the differences between B-type and A-type interfaces. Our results highlighted that lateral communication is altered at the seam and that residues involved in the lateral coupling of PFs are on average less conserved than those coupling tubulins along the PF. Taken together, these results provide useful insights to understand the mechanical communication within the MT and how its alteration can affect the stability of PF interactions.

## Methods

### Preparation and simulation of the models

To study tubulin dynamics within the MT, we employed patches of the MT wall. The structure of GDP-bound MT was retrieved from the Protein Data Bank [PDB code 3J6F (Alushin et al. 2014)] and used to build the models. Since the selected structure is not composed of human tubulin, homology modelling (2019) was employed to build a sheet of human $\alpha$ Ib-$\beta$ III tubulins (see Sect. 1.1 of Supplementary Material). The βIII isotype has been selected since it is the isotype with the greatest increase in expression in tumoral tissues compared to healthy phenotypes and thus represents a promising target for cancer treatment (Leandro-García et al. 2010; Tseng et al. 2010; Yeh et al. 2016; Wang et al. 2017; Pallante et al. 2020), whereas the αIb isotype was chosen because it is known to assemble mainly into 13 PF MTs together with βIII tubulin (Ti et al. 2018). The thus obtained system is named S1 in the following. To account for the presence of the seam and study possible differences between A-type and B-type interfaces, two additional systems, named herein S2 and S3, were created by inverting the position of $\alpha$ and $\beta$ tubulins at the sides of the sheet. Therefore, in system S1 the central tubulin dimer forms B-type contacts on both sides and models the tubulins far from the seam, while in systems S2 and S3 the dimer has one B-type and one A-type contact and models tubulins at the seam (Fig. 1). Then, the same simulation protocol (see Sect. 1.2 of Supplementary Material) has been subsequently followed to obtain three 500 ns long MD replicas for each system. All simulations were carried out in GROMACS 2021.4 (Lindahl et al. 2021).

### Analysis

We visually inspected the MD trajectories using the visual molecular dynamics (VMD) suite (Humphrey et al. 1996). The MD simulations of each system were analysed as follows. We first quantified the structural stability of the system by computing the root-mean-squared deviation (RMSD) of backbone positions from their initial coordinates. The trajectories at equilibrium were concatenated to perform further analyses (see Sect. 1.3 of Supplementary Material). Briefly, the coupling between the central tubulin and the adjacent ones was described in terms of frequency of contacts (Cannariato et al. 2022) and frequency of specific interactions (e.g., hydrophobic interaction, salt bridge) (Adasme et al. 2021; Miceli et al. 2022). The mechanical properties at the single-residue level were investigated by computing the force constant (Navizet et al. 2004; Lavery and Sacquin-Mora 2007; Cannariato et al. 2022). The structural communication within the tubulin sheet has been inferred through the dynamical network analysis approach, using the dynetan library (Melo et al. 2020), since it takes into account also the nonlinear contribution to amino acid dynamical correlations. The network analysis of MD simulations was employed as it is recognized as a powerful technique to infer global network parameters that characterize protein dynamics (Melo et al. 2020), allowing to obtain information about how molecular vibrations propagate inside a protein structure through interacting amino acids combining information about system structure and dynamics. The network was characterized in terms of the degree of centrality and betweenness centrality. The degree of centrality is defined as the number of edges of a node and can be interpreted as a measure of local influence for the specific node at the local level. It is computed as:

$$d{g}_{i}=\sum_{i\ne j}{A}_{ij}{w}_{ij}$$

where *A* is the adjacency matrix that defines the connection in the graph, and *w*_*ij*_ is the weight associated with the edge between node *i* and *j*, i.e. the generalized correlation coefficient. The betweenness centrality of a node or edge quantifies the number of shortest pathways in which a specific node or edge takes part, therefore is useful to highlight nodes and edges important for the connection of distant parts of the network. It is computed as:

$${b}_{i}=\frac{1}{C}\sum_{s,t \in V}\frac{\sigma \left(s,t|i\right)}{\sigma \left(s,t\right)}$$

where *V* is the set of nodes of the graph, $\sigma \left(s,t\right)$ is the number of shortest paths between nodes *s* and *t*, $\sigma \left(s,t|e\right)$ is the number of these paths that pass through node *e*, and *C* is a normalization factor that, in a network of *n* nodes, is equal to

$$\frac{2}{(n-1)(n-2)} \mathrm{or }\frac{2}{n(n-1)}$$

for node and edge betweennesses, respectively. In this analysis, the shortest path between two nodes is the one that maximizes the sum of correlations between nodes involved in the path. It is worth mentioning that the tubulins on which position restraints were applied during the MD simulations were not included in the network analysis. Finally, the evolutionary conservation of tubulin residues has been defined by computing the normalized stereochemically sensitive Shannon’s entropy (NE) from multiple sequence alignment (MSA) (Mirny and Shakhnovich 1999; Valdar 2002).

## Results

The RMSD plots showed that the structural stability of the system on each replica was reached after the first 100 ns (Fig. S2). The structural stability was confirmed by a cluster analysis of the last 400 ns, using the linkage algorithm, the RMSD between the alpha carbons of the central tubulin heterodimer as metric, and a cutoff of 0.15 nm, which showed only one cluster per simulation replica. Then, we analysed the trajectories of S1, S2, and S3 as follows. First, we analysed the systems through the network approach to characterize communication inside the tubulin sheet. Then, we inspected the interactions between vertically and horizontally coupled tubulins, with specific attention to changes at the seam. Finally, we coupled our results with the analysis of amino acid conservation to understand whether key residues for structural communication are evolutionarily conserved.

### Dynamical network analysis

**Fig. 2:**
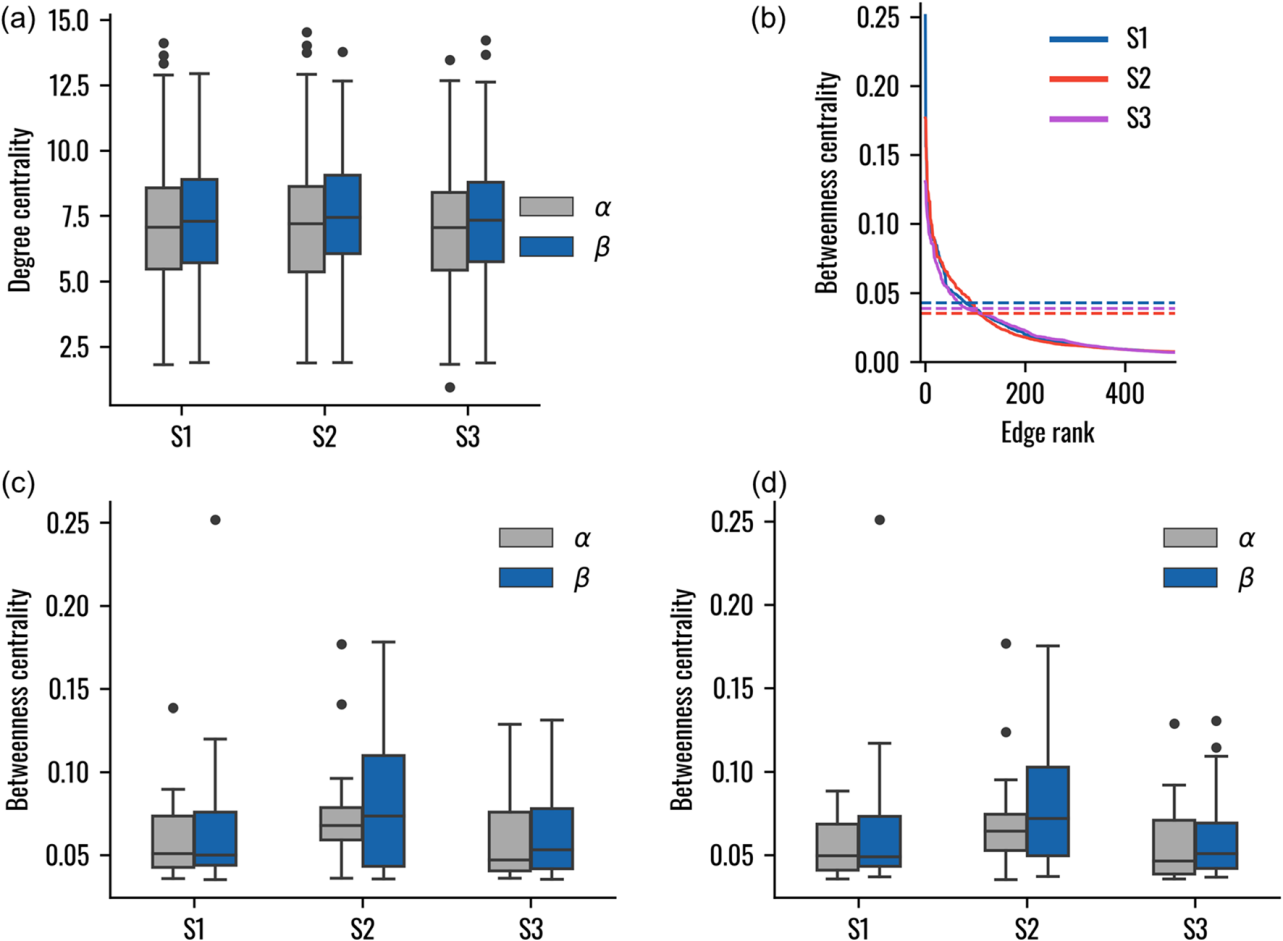
Network metrics.** a** Boxplot showing the degree of centrality for the central tubulin heterodimer nodes. **b** Ranked betweenness centrality for the edges having a node belonging to the central tubulin heterodimer in at least one extremity. The horizontal lines represent the value at which a knee was identified in the three systems. **c** Boxplot showing the betweenness centrality of the central tubulin heterodimer nodes. Values under the identified threshold are not included. **d** Boxplot showing the betweenness centrality of the edges having a node belonging to the central tubulin heterodimer in at least one extremity. Values under the identified threshold are not included

The dynamical networks representing the three systems were first analysed in terms of degree of centrality, highlighting similar local influence for *α* and *β*-tubulin residues (Fig. 2a). Plotting the edge betweenness centrality versus the edge rank revealed that the betweennesses in the three networks had similar values, with the only differences being observed in the few top ranking edges (Fig. 2b). The knee of the curve was identified in the three systems and the lowest one, belonging to S2, was used as threshold in the subsequent analysis. In particular, the distribution of edges and nodes betweenness showed that the *β*-tubulin is characterized by a tendency towards higher centrality independently of the system (Fig. 2c, d). Therefore, the edges and nodes with highest betweenness tend to involve more *β*-tubulin both in the presence and in the absence of the seam.

**Fig. 3:**
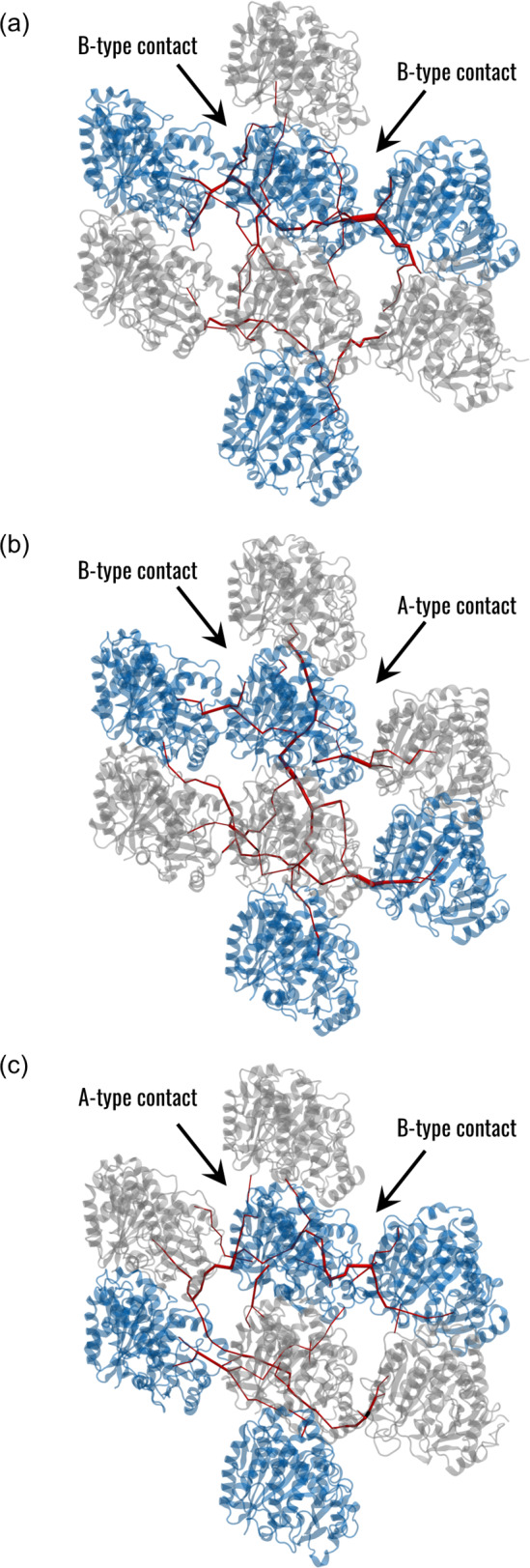
Analysis of structural communication in the three systems. Visual representation of the edges with betweenness centrality higher than the identified threshold (red) obtained in the (**a**) S1, (**b**) S2, and (**c**) S3 systems. The $\beta$-tubulin is represented in blue and the $\alpha$-tubulin in grey. The width of the edges is proportional to their betweenness

Interestingly, the localization of the top ranking edges in the network highlights differences in the three systems. In S1, a clear horizontal pathway linking the *β*-tubulins could be observed, while a less central pathway was horizontally connecting the *α*-tubulins (Fig. 3a). Among the top ranking edges at the interface between neighbouring tubulins, the two with the highest betweennesses were connecting *β*-tubulins, while lower values were observed for *α*-tubulins. In S2, a vertical pathway along the PF was observed together with the horizontal paths and the two interface edges with the highest centrality were located at the A-type contact (Fig. 3b). Finally, in S3, two horizontal pathways were observed connecting *α* and *β*-tubulins and converging at the A-type contact (Fig. 3c). In this case, the two interface edges with the highest betweenness were placed at the B-type interface. The difference in the mechanical communication at the B-type and A-type interfaces was then characterized by focusing on the connections at such interfaces and their centralities, as reported in Table S1. In general, residues forming highly central connections were located in the M-loop, H1’-S2 loop, H3, and H9 regions for both tubulins, while the involvement of one amino acid in H10 was observed in α-tubulin. It is worth noticing that, independently of the system, the most central edges were located at the M-loop side of the central tubulin. Therefore, regarding the betweenness centrality, the communication between tubulins in regions far from the seam is not symmetric. Moreover, the presence of a structural discontinuity such as the seam induces a different communication, which in turn depends on the location of the seam.

### Interactions between neighbouring tubulins

**Fig. 4:**
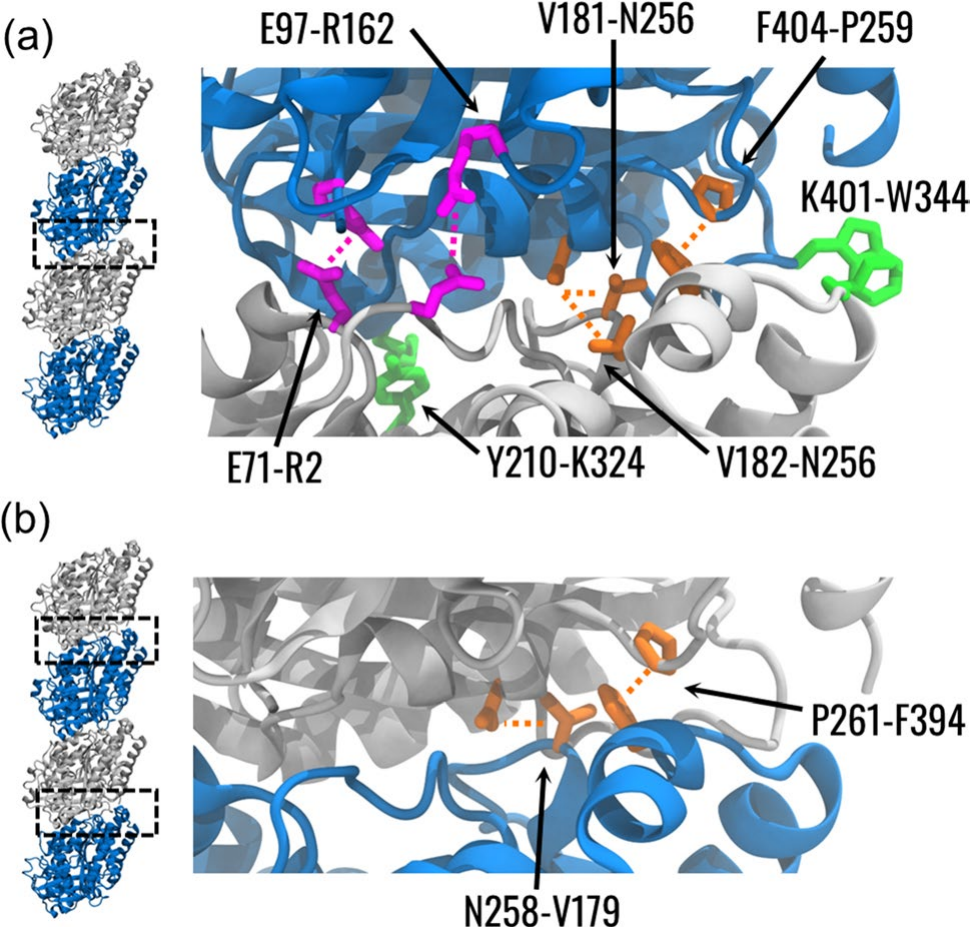
Analysis of interaction between vertically coupled tubulins. Visual representation of the residues pairs forming stable interaction at the $\alpha \beta$-interface (**a**) within a tubulin dimer and (**b**) between two tubulin dimers. Residues are coloured according to the type of interaction as identified by PLIP, with salt bridges, hydrophobic interactions, and hydrogen bonds rendered in magenta, green, and orange, respectively. Labels are formatted as [$\alpha$ residue]-[$\beta$ residue]. The dashed lines show the interacting residues without indicating the specific atoms involved in the interaction

To investigate the differences between the intra-dimer and the inter-dimer surfaces, we identified the nature of stable interactions at these interfaces. An interaction was defined as stable if the mean frequency across the three systems was greater than 0.75. At the intra-dimer interface, 3 hydrogen bonds, 2 hydrophobic interactions, and 2 salt bridges were detected (Fig. 4a). Among them, the *α*E97-βR162 salt bridge and the *α*Y210-βK324 hydrophobic interaction corresponded to edges with betweenness higher than the threshold. Some difference was identified at the inter-dimer interface, where 2 hydrogen bonds were highlighted (Fig. 4b). Notably, this analysis showed a highly stable hydrogen bond between αF404 and βP259 at the intra-dimer interface, and a corresponding interaction between αP261 and βF394 at the inter-dimer interface. Interestingly, these interactions were also the most stable among the identified ones (Fig. S3a).

**Fig. 5:**
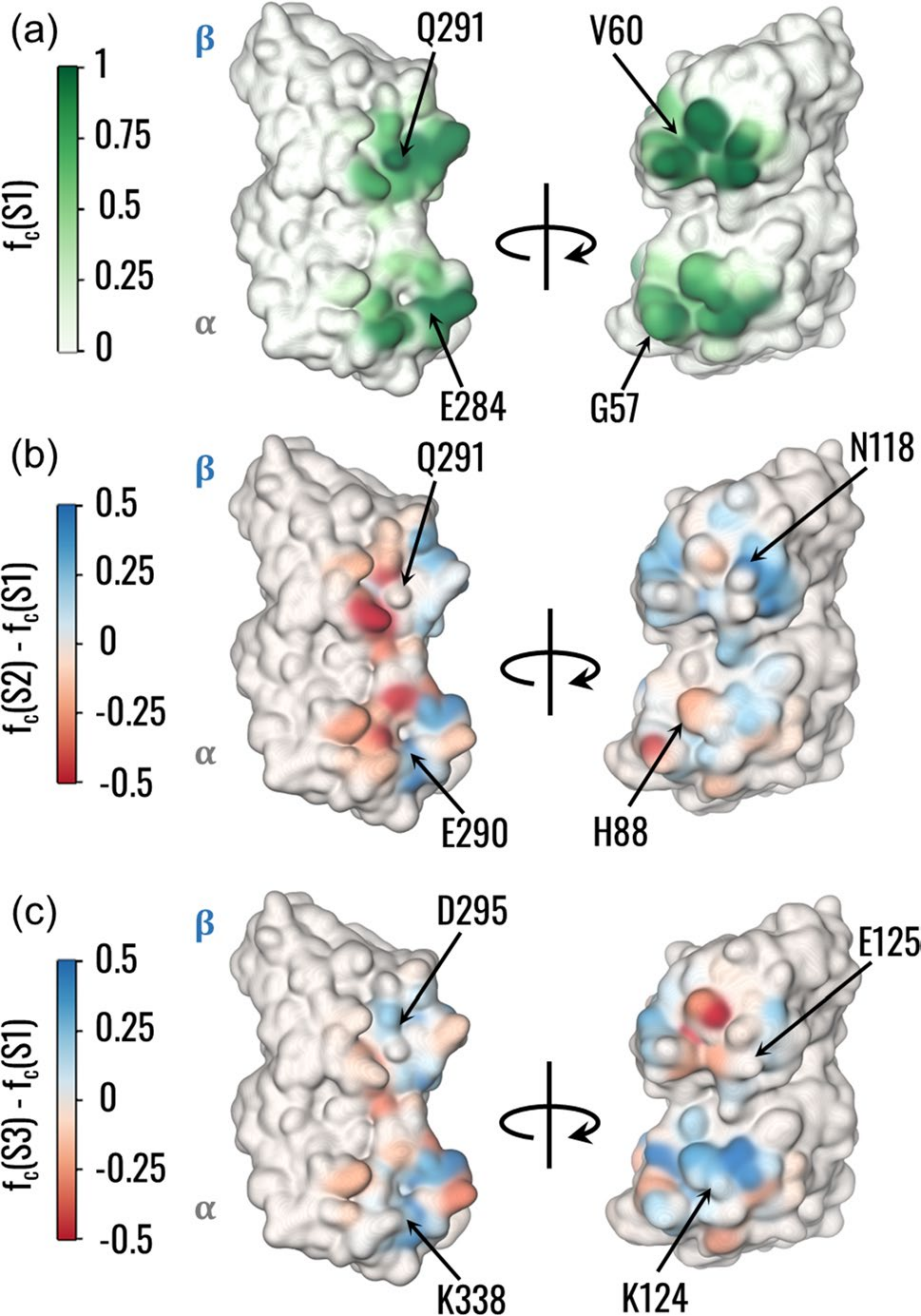
Difference in the lateral contacts between A-type and B-type interfaces. **a** Visual rendering of the frequency for the central dimer to be in contact with the adjacent tubulins. Residues involved in edges with the highest betweenness on α and β tubulins are pointed out. **b**,** c** Visual rendering of the difference between the contact frequency in S2 (**b**) or S3 (**c**) and S1. Residues involved in edges with the highest betweenness on α and β tubulins are pointed out

Based on the structural communication between PFs, we analysed the frequency of contacts between the central heterodimer residues and adjacent tubulins. Consistently with network analysis, H3 and H9 regions together with the M-loop and H1’-S2 loop were mainly involved in the contacts. Within S1, regions with high contact frequencies were wider in the $\beta$-tubulins. Moreover, the most central amino acids were characterized by high contact frequencies (Fig. 5a). Similar frequency distributions were obtained in S2 and S3, with the highest differences from S1 located at the seam. In detail, the A-type interface of S2 was characterized by decreased frequency in *β*H10 region and increased contacts in the M-loops of both tubulins (Fig. 5b). In the S3 seam, there was a loss of contacts in *β*V60 and βD88, while increased frequency was observed in the corresponding residues of *α*-tubulin, together with an increase of contacts in *α*H3 region (Fig. 5c). On the other hand, reduced differences in the contact frequency were observed at the B-type interface, with a moderate increase in *β*H3 region (S2), βH9 helix, and *α*M-loop (S3). In addition to the distribution of contacts, an analysis of specific interactions involving amino acids of the central tubulins was performed, highlighting that stable interactions identified by PLIP are mainly salt bridges at both types of interfaces (Fig. S3b). Interestingly, only a few of the residues forming stable interactions were pinpointed by the network analysis. However, alterations in their interaction frequencies were not directly linked to the changes pointed out by the network analysis.

### Evolutionary and mechanical analysis of tubulin

**Fig. 6:**
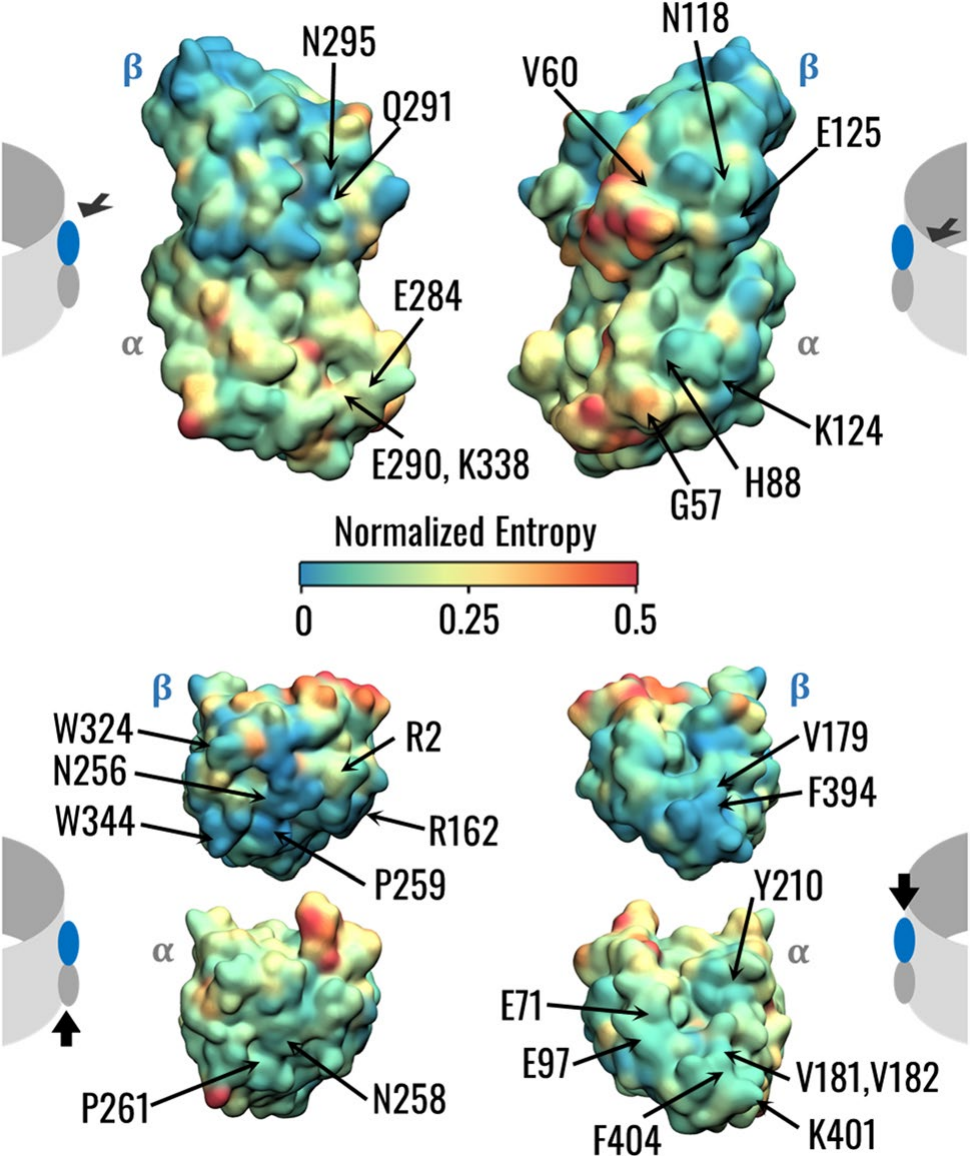
Evolutionary conservation of $\alpha$ and $\beta$-tubulins. Visual representation of the tubulin dimer surface coloured according to the conservation score. The different views are specified by arrows in the cartoons at the left and right sides of the figure, showing a tubulin dimer inserted in an MT wall. Key residues highlighted by the network and PLIP analysis are pointed out

The MT lattice features are related to the organism of expression and tubulin isotype (Chaaban and Brouhard 2017; Ti et al. 2018). In this context, the computation of the so-called normalized entropy (NE) together with the network analysis may help to shed light on the relationships between the evolutionary conservation of tubulin residues and their role in microtubule stability. Results in terms of NE distribution showed that, on average, the $\beta$-tubulin is more conserved than the $\alpha$-tubulin, however, both sequences are highly conserved, in line with previous studies (Little et al. 1981; Ludueña 2013) (Fig. S4). To investigate the relationship between structural communication and evolutionary conservation, we visualized the NE distribution on the tubulin surface (Fig. 6). The regions characterized by the lowest conservation are located in corresponding regions of the $\alpha$ and $\beta$-tubulins, in particular the H1-S2 loop which was also highly flexible in $\alpha$-tubulin during the simulations (Fig. S5). Interestingly, not all residues involved in the interaction with adjacent PFs are highly conserved, such as $\beta$-tubulin N126 and R282. On the contrary, the upper and lower surfaces of tubulins, involved in interactions within the PF, are more conserved than the lateral ones.

**Fig. 7:**
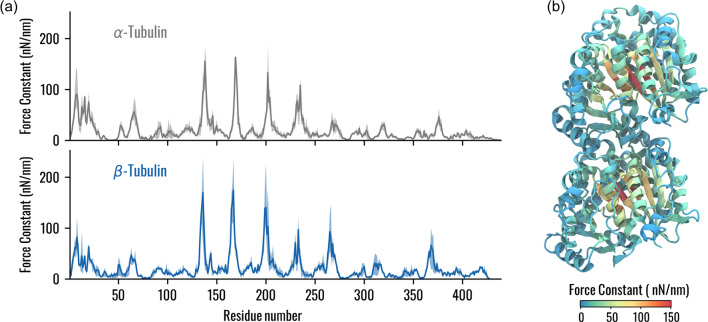
Identification of mechanically rigid residues. **a** Force constant for $\alpha$ and $\beta$-tubulins. The average value of the three systems is represented with a continuous line, while the shaded region highlights the minimum and maximum values obtained. **b** Visual representation of a tubulin dimer with residues coloured according to the mean force constant

The identification of mechanically rigid residues has been performed through the computation of the force constants and, as done in previous literature, the relationship between mechanical rigidity and conservation has been investigated (Navizet et al. 2004; Felline et al. 2019). In general, considering only the amino acids with a force constant greater than the median of the profile, the median NE for α and β-tubulin showed that the conservation of mechanically rigid residues is higher for $\beta$-tubulin. Such values are lower than the median value of the NE distribution over all tubulin residues, meaning that amino acids with high force constant are mainly conserved (Fig. S6). Moreover, the analysis of the force constant profile showed four main peaks in the corresponding regions of the two tubulins. In addition to these common features, one additional peak was observed in $\beta$-tubulin (Fig. 7a). The identified residues are located in the central $\beta$-sheet of tubulins (Fig. 7b), highlighting the importance of this structured region in the mechanical stability of tubulin. Interestingly, some amino acids forming peaks of the force constant profiles are not highly conserved (Fig. S7), meaning that rigid points of the tubulin structure may have been mutated during evolution without altering the mechanical stability of tubulin within the MT lattice.

## Discussion

Although the pivotal role of MTs for cell functions is undoubtedly recognized, mechanisms and features related to MT's unique properties are still a matter of debate in the literature (Prosser and Pelletier 2017; Ichikawa and Bui 2018; Nachury and Mick 2019; Vicente and Wordeman 2019). MT architecture, strongly related to mechanical behaviour, has been finely optimized by nature depending on the functions to be accomplished (Tuszyński et al. 2005). Therefore, the tubulin structure and isotype have been tuned during evolution to optimally assemble and mechanically communicate within the MT structure. This might suggest that MT targeting agents can alter the structural communication between tubulins to stabilize or destabilize the MT architecture. While in previous literature the vibration modes and consequent deformation patterns at the tubulin level were investigated to relate the tubulin-level properties to the overall mechanics of the MT (Deriu et al. 2010; Havelka et al. 2017), in this work, we focused on a smaller system and analysed it by extensive MD simulations and dynamical network analysis to provide a more dynamic description of tubulin communication inside the MT lattice. Given that the understanding of tubulin association in the MT and how vibrations might propagate inside such assembly, also due to its natural vibrations, is still incomplete, this work provides novel insights into the mechanical communication within the MT wall. Although not providing an experimental proof of communication, this modelling approach is useful to support experimental activity and understand phenomena at a molecular resolution (Angelova et al. 2011). From the analysis of interactions between vertically coupled dimers, it was observed that the tubulin abluminal region is involved in the formation of stable hydrogen bonds both at inter-dimer and the intra-dimer interfaces (Fig. 4). Moreover, one stable hydrogen bond interaction between a proline and a tryptophane, located next to helix H8, was observed both at the inter-dimer and the intra-dimer interfaces. Interestingly, H8 of $\beta$-tubulin has been previously identified as an anchor point for the bending motion of the tubulin heterodimer, which was suggested to regulate tubulin binding into the MT lattice (Igaev and Grubmüller 2018). Moreover, the same region in $\alpha$-tubulin was identified to be involved in an alteration of MT lattice after GTP hydrolysis (Alushin et al. 2014; Zhang et al. 2015). Therefore, our results strengthen previous findings regarding the importance of the abluminal region of tubulin in the MT stability and highlight central interactions for the stability within the PF.

Regarding the structural communication within the MT lattice, several studies have emphasized that the A-type contacts are weaker than the B-type ones (Sept et al. 2009; Igaev and Grubmüller 2022). Indeed, it has been observed that the presence of A-type interfaces in the MT lattice destabilizes inter-PF interactions and, consequently, the MT architecture (Katsuki et al. 2014). In this context, our results from the network analysis show that the structural communication is altered by the presence of an A-type contact (Fig. 3). In particular, the transfer of information about mechanical vibrations seems to be transduced more by *β*-tubulins than *α*-tubulins. Notably, the key residues involved in the such transduction involve the M and H1’-S2 loops, which were already identified to be involved in the inter-PFs interaction by previous studies (Sui and Downing 2010). Interestingly, the edges located at the M-loop side of the central heterodimer had higher betweenness values independently of the system, suggesting an asymmetry or directionality of the communication. In line with this observation, the different location of the seam in S2 and S3 systems resulted in different communication patterns (Fig. 3). At the same time, not all the highly central residues at the interface between adjacent PFs were the most conserved (Fig. 6). These results might be relevant to explain the reason behind β-tubulin isotype controlling the MT architecture in terms of the number of PFs and why different architectures had been found in different organisms (Chaaban and Brouhard 2017; Ti et al. 2018; Janke and Magiera 2020). Moreover, this study could be related to previous findings regarding the cooperative binding of kinesin on MT, which observed that kinesin binding increases the binding affinity towards kinesin beads over micrometres in MT length (Muto et al. 2005; Wijeratne et al. 2022). In fact, the methodological approach proposed in this work has the potential to investigate how the conformational change induced to tubulin by kinesin binding (Krebs et al. 2004) is propagated inside the MT lattice to originate a long-range increase in kinesin binding affinity along the MT. On the other hand, we observed that the abluminal region of tubulins, involved in the formation of stable hydrogen bonds in both the intra-dimer and inter-dimer interfaces, was highly conserved, which is coherent with the fact that, in all MT architectures, the tubulin heterodimers assemble vertically to form PFs. Moreover, the peaks of the force constant profile were located in one central $\beta$-sheet, suggesting a pivotal role of this structure within tubulin, although some residues with high force constant were not the most conserved (Figs. 7, S5, 6).

We also investigated the differences in lateral coupling of PFs at the seam that might explain the obtained communication pathways. The analysis of contact probabilities was coherent with previous literature showing that M, H1’-S2, and H2-S3 loops were involved in a lock-and-key configuration (Sui and Downing 2010; Ti et al. 2018). Our dynamical analysis showed that loss of contacts for $\beta$-tubulin and local increases in the contact frequencies for $\alpha$-tubulin were observed at the seam (Fig. 5a–c). At the same time, we observed the change in the structural communication observed at the seam was related to a change in the distribution of contact probabilities at the lateral interface rather than in the stability and nature of the interaction involving crucial residues in the pathway. Thus, our results show that the reduced stability of PFs coupling at the seam can be related to changes in the distribution of contacts, leading to an altered structural communication.

In conclusion, our work combines MD to dynamic network analysis to investigate propagation of mechanical vibrations through the MT wall. It is worth noticing that, to maintain the correct geometry characterizing the MT wall, position restraints have been applied to the four vertices of the simulated MT wall sheet, as described in the Method section. This might have had influenced the dynamics of tubulin residues close to position restrained regions. However, those region are far from and not interacting with any region of interest for conformational and dynamical network analysis (as explained in Sect. 1.2 of Supplementary Material). On the other hand, this work has enlighted how mechanical perturbation may propagate throughout the MT supramolecular assembly showing local to global correlations characterizing the structural and dynamical network architecture. In this concern, the proposed approach has the potential to highlight hidden structural communication pathways in biofilaments, thus providing useful insights for the study of subcellular mechanics, the structure-based design of drugs, and the characterization of bioinspired materials (Gentile et al. 2015; Nepal et al. 2022).

## Conclusions

In the present work, a computational analysis of the structural communication within the MT lattice has been performed by means of molecular modelling techniques. Starting from the all-atom MD simulation of MT sheets, both containing the seam and with only homophilic interactions, we identified how mechanical information about amino acid vibrations is transmitted across the MT wall. Our results show that the seam represents a discontinuity in the lateral communication pathway, which is likely to be controlled mainly by $\beta$-tubulins. Therefore, this study provides novel insights into the molecular basis of tubulin association in MTs and how the alteration of the communication pathways might result in weaker interfaces. While representing an initial study on the modelling of tubulin coupling in the MT lattice, the presented methodology could be extended to study a number of states concerning GTP hydrolysis (GMPCPP, GDP + Pi, GDP, and GDP) and the presence of anticancer drugs such as Taxol, to fully characterize the structural communication inside a stabilized MT lattice. Indeed, a better understanding of relationships between structure, mechanics, and MT functions, is also important for medical applications, since MT targeting agents are broadly used and studied in cancer therapies (Leung et al. 2015; Kellogg et al. 2017; Pallante et al. 2020). Moreover, it is well known that the GTP-bound MT is characterized by more stable inter-protofilament interactions and that lateral contacts are weakened with GTP hydrolysis, leading to MT catastrophe (Igaev and Grubmüller 2018, 2022; Manka and Moores 2018). A better overall comprehension of tubulin association and communication in the MT might be useful not only for the computational design of MT stabilizing and destabilizing agents for cancer treatment but also in the design of hierarchical bioinspired materials (Nepal et al. 2022).

### Supplementary Information

Below is the link to the electronic supplementary material.

Supplementary file1 (PDF 710 kb)

## Data Availability

All data to replicate the simulations are accessible at 10.5281/zenodo.10246255.
