# Supplementary material for: Mechanical communication within the microtubule through network-based analysis of tubulin dynamics
Source: Biomech Model Mechanobiol. 2023 Dec 7;23(2):569–79. doi: 10.1007/s10237-023-01792-5 (PMC10963519; doi:10.1007/s10237-023-01792-5)
Supplement: Supplementary file 1 — Supplementary file1 (PDF 710 kb) [file 10237_2023_1792_MOESM1_ESM.pdf]

Mechanical Communication within the Microtubule through Network-based Analysis of Tubulin Dynamics

Marco Cannariato[1], Eric A. Zizzi[1], Lorenzo Pallante[1], Marcello Miceli[1] and Marco A. Deriu[1*]

[1] Polito^{BIO}Med Lab, Department of Mechanical and Aerospace Engineering, Politecnico di Torino, Turin, Italy

*Corresponding author. e-mail: marco.deriu@polito.it

Supplementary Information

1 Methodological details

1.1 Preparation of the model

Starting from the structure of GDP-bound MT (PDB code 3J6F (Alushin et al. 2014)), the upper and lower three tubulins were removed to obtain a patch of 3x4 tubulins, which will be referred to as "sheet" in the following. Homology modelling was employed to build a sheet of human αIb-βIII tubulins. The amino acid sequences were retrieved from the UniProtKB database, considering residues 1-439 for α tubulin (accession code P68363) and 1-429 for β tubulin (accession code Q13509). Then, each monomer was modelled using the Molecular Operating Environment (MOE) software (2019) version 2020; GDP, GTP, and magnesium positions were maintained from the original crystal structure.

1.2 Molecular Dynamics

The same simulation protocol has been subsequently followed for each system. The protonation state of the protein at a pH of 7.4 was predicted with PROPKA (Olsson et al. 2011), and its simulation parameters were obtained using the AMBER ff99SB-ILDN force field (Lindorff-Larsen et al. 2010). Topologies for the GDP and GTP ligands were built through the General Amber Force Field (GAFF) (Wang et al. 2004), using the AM1-BCC method (Jakalian et al. 2002) to assign partial charges, as done in previous literature (Gentile et al. 2015; Pallante et al. 2020). The system was centred in a cubic box of dimensions around 20 nm x 18 nm x 9 nm, which was solvated using explicit TIP3P water. Finally, Na^+ and Cl^- ions were added to neutralize the net charge and to reach a physiological salt concentration of 0.15 M. Energy minimization was performed through 1000 steps of the steepest descent algorithm. Then the following procedure was followed to produce three distinct MD replicas for each system: first, temperature coupling was achieved by a 200 ps-long simulation in the NVT ensemble, using the modified Berendsen thermostat (Berendsen et al. 1984) at a reference temperature of 300 K (τ = 1 ps). Then, pressure coupling was obtained through a 500 ps-long simulation in the NPT ensemble, using the Berendsen barostat at a reference pressure of 1.0 bar (τ = 5 ps). In both these equilibration simulations, the positions of all alpha carbons (Cα) were restrained using a force constant of 1000 kJ/mol nm^2. Finally, a 500 ns-long MD simulation was produced in the NPT ensemble with a 2 fs timestep using the leapfrog algorithm. During this simulation, a position restraint was applied to the Cαs of one β-sheet of the tubulins at the four vertices of the sheet (residues 132-138, 163-170, and 198-203 for β-tubulins and 133-140, 165-171, and 200-204 for α-tubulins) (Fig. S1). This approach ensured to avoid an excessive deviation, during the simulation, of the experimental sheet curvature, which is representative of the MT wall, without restraining the tubulins whose dynamics were analyzed. Electrostatic interactions were treated using the Particle Mesh Ewald

method, with a short-range cutoff of 1.2 nm, and potential switching starting at 1.0 nm. Van der Waals interactions were treated with a short-range cutoff at 1.2 nm and potential switching starting at 1.0 nm.

1.3 Simulation analysis

The MD simulations of each system were analyzed as follows. The structural stability of the system was computed by computing the root-mean-squared deviation (RMSD) of backbone positions, both of the central tubulin heterodimer and the whole sheet, from their initial coordinates. The trajectories at equilibrium were concatenated to perform further analyses. All analyses were carried out by sampling the concatenated trajectory every 50 ps unless otherwise specified. The most mobile regions were identified by analyzing the root-mean-squared fluctuations (RMSF) of Cα atoms.

The frequency of contacts between each residue of the central tubulin and the adjacent tubulin was determined according to previous literature (Cannariato et al. 2022). Briefly, for each MD trajectory frame, considering a sampling interval of 250 ps, the minimum distance between each residue (also considering hydrogen atoms) of the central tubulin dimer and the lateral tubulins has been computed: a residue was considered to be in contact if the minimum distance was below the threshold of 0.42 nm. The chosen threshold allows to consider also interactions characterized by higher inter-atomic distances, such as hydrophobic interactions. The final contact frequency for a given residue is obtained by dividing the number of frames in which such residue was in contact by the total number of considered frames.

The frequency of a specific interaction (e.g., hydrophobic interaction, salt bridge) between each central tubulin residue and the adjacent tubulins has been computed by inferring the interaction through the PLIP software (Adasme et al. 2021) on each frame, sampling every 250 ps, and then averaging the number of the occurrences on the total number of frames (Miceli et al. 2022).

The mechanical properties at the single residue level were investigated by computing the force constant, which is a measure of the deviation of the mean distance of a residue from the rest of the protein, as done in previous literature (Navizet et al. 2004; Lavery and Sacquin-Mora 2007; Cannariato et al. 2022). The force constant was computed separately for the α and β tubulins of the central heterodimer according to the formula

$$k_i = \frac{3k_BT}{\langle (d_i - \langle d_i \rangle)^2 \rangle},$$

where k_B is the Boltzmann's constant, T is the temperature (300K), d_i is the mean distance of the i-th residue from the rest of the protein, considering the Cα, and the $\langle\ \rangle$ operator indicates a mean over the trajectory.

The structural communication within the tubulin sheet has been inferred through the dynamical network analysis approach, using the dynetan library (Melo et al. 2020). In detail, in the dynamical network analysis, each protein residue was represented by a node located in its C-alpha, GTP and GDP were modelled through a single node in the closest atom to their centre of mass, and magnesium ions were represented by a single node. According to previous literature (Melo et al. 2020), nodes were connected with edges if the corresponding residues were in contact for more than 75% of the simulation time, where two residues are in contact at a certain time if the shortest distance between heavy is shorter than 4.5 Å. The edges of the obtained graph were weighted using the generalized correlation coefficient (Lange and Grubmüller 2005) to include dynamic information inside the network. For this purpose, the concatenated equilibrium trajectories were considered as done previously (Manrique et al. 2023).

RMSD and RMSF were computed through the GROMACS analysis suite. Force constant, contact frequency, and interaction frequency were computed through custom-made python scripts and the MDAnalysis library (Gowers et al. 2016).

1.4 Evolutionary conservation

The evolutionary conservation of tubulin residues has been defined by computing the normalized stereochemically sensitive Shannon's entropy (NE) from multiple sequence alignment (MSA) (Mirny and Shakhnovich 1999; Valdar 2002). In detail, the amino acid sequences of α and β tubulins were downloaded from UniProt, for a total of 83 sequences for α tubulin and 120 for β tubulin. MSA was performed in MOE, and then the conservation score of the i-th residue was computed as:

$$NE_i = \frac{-\sum_{j=1}^{k} p_i(j) \log_2 p_i(j)}{\log_2 N},$$

where $k = 7$ is the number of residue classes, as defined in previous literature and including the gap (Mirny and Shakhnovich 1999), N is the number of sequences in the MSA, and $p_i(j)$ is the probability of finding the j-th class at the i-th position. Due to normalization, NE ranges from 0, meaning totally conserved, to 1, meaning non-conserved. NE calculation was implemented in custom-made python scripts.

2 Supplementary Figures

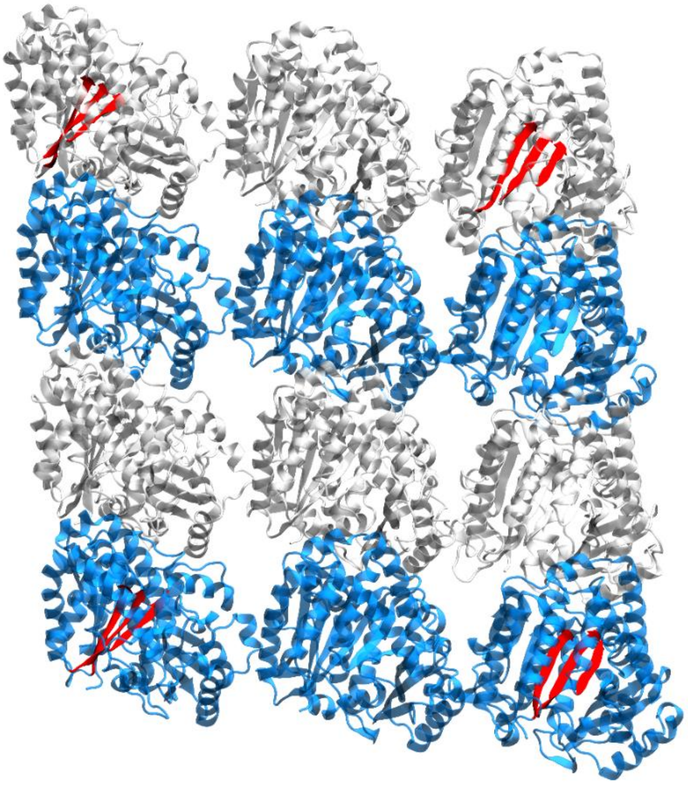

Fig. S1 Visual rendering of the position restraints applied to the system. Residues with position restraints applied are coloured in red. System S1 is depicted as an example, and the same approach was followed in all systems under analysis

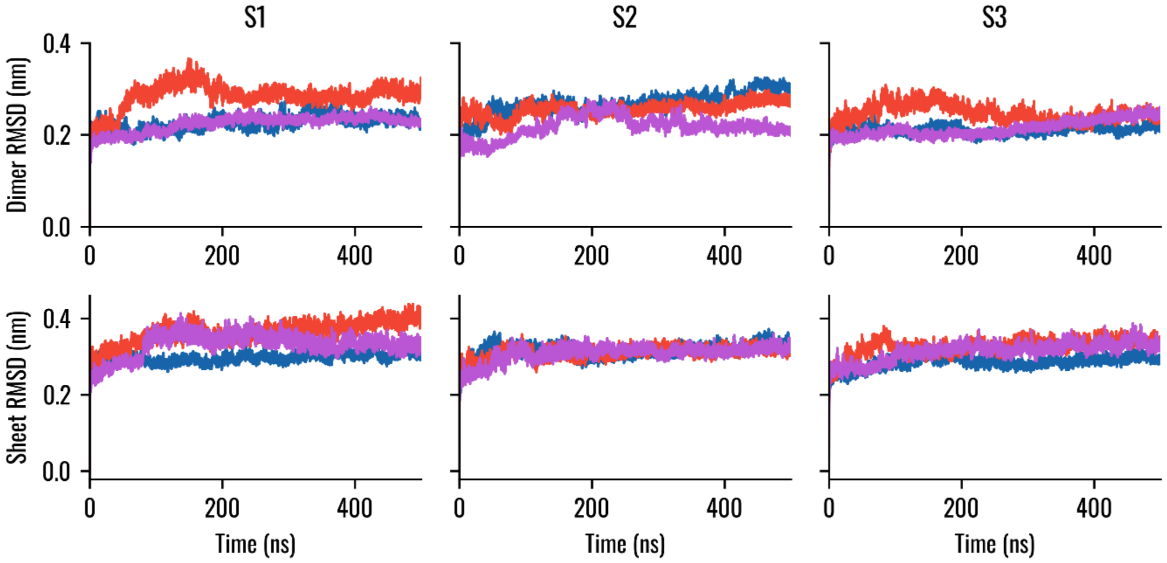

Fig. S2 Simulations convergence. Root-mean-squared deviations, computed on the protein backbone after least-square fitting on the protein backbone, of the central tubulin heterodimer (top) and the tubulin sheet (bottom) from its initial position in the three systems. Different colors represent the different replicas

Table S1 Top ranking edges adges at the lateral interface between neighboring tubulins. The table reports the tubulin, residue name, residue number, and normalized entropy of the nodes connected by the edge. Betweenness value, type of contact, and side of the interface (relative to the central heterodimer). The first node of the edge always belongs to the central tubulin and edges are sorted by betweenness value

	Tub1	Name1	Num1	NE1	Tub2	Name2	Num2	NE2	Btw	Type	Side
**S1**	β	GLN	291	0.09	β	LYS	122	0.09	0.251087	B	M
	β	VAL	60	0.06	β	TYR	281	0.02	0.100414	B	H1-S2
	α	GLU	284	0.16	α	GLY	57	0.29	0.088339	B	M
	α	GLY	57	0.29	α	GLN	285	0.17	0.085034	B	H1-S2
	β	ASP	88	0.04	β	ARG	282	0.22	0.076651	B	H1-S2
	β	GLU	125	0.04	β	GLN	291	0.09	0.044085	B	H1-S2
	α	ASP	127	0.03	α	LYS	338	0.14	0.040793	B	H1-S2
**S2**	α	GLU	290	0.16	β	GLU	125	0.04	0.17676	A	M
	β	GLN	291	0.09	α	ASP	127	0.03	0.147382	A	M
	β	ASP	118	0.07	β	ASP	295	0	0.099778	B	H1-S2
	α	HIS	88	0.09	α	HIS	283	0.12	0.076305	B	H1-S2
	α	THR	56	0.18	α	GLU	284	0.16	0.075635	B	H1-S2
	α	VAL	62	0.08	α	HIS	283	0.12	0.067484	B	H1-S2
**S3**	β	ASP	295	0	β	ASP	118	0.07	0.130601	B	M
	α	LYS	338	0.14	α	ASP	127	0.03	0.128851	B	M
	β	GLU	125	0.04	α	LYS	338	0.14	0.114571	A	H1-S2
	α	LYS	124	0.07	β	ASP	295	0	0.091994	A	H1-S2
	β	ARG	282	0.22	β	ARG	86	0.13	0.055578	B	M
	α	HIS	88	0.09	β	SER	278	0.19	0.055014	A	H1-S2
	β	LYS	58	0.15	α	HIS	283	0.12	0.046663	A	H1-S2
	α	HIS	88	0.09	β	ARG	282	0.22	0.036523	A	H1-S2

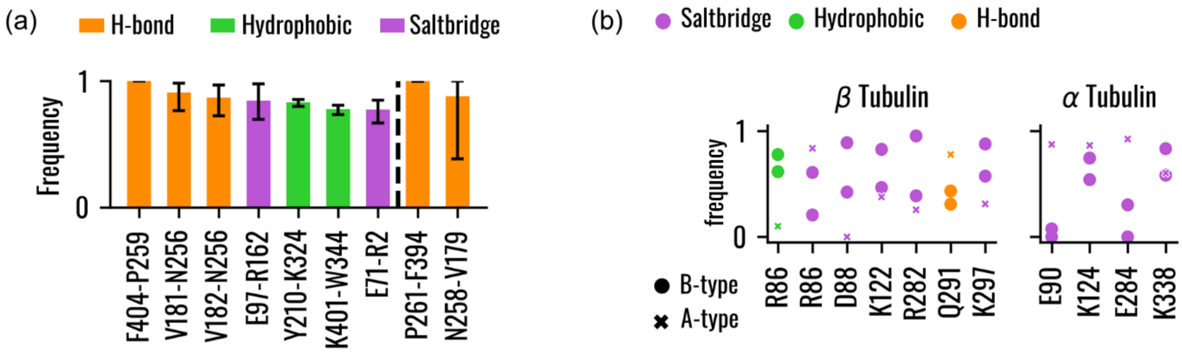

Fig. S3 Frequencies of specific interactions. **a** Interaction frequency between the residue pairs identified as stable. Bars represent the mean interaction frequency, while error bars correspond to the minimum and maximum frequencies across the three systems. The dashed lines divides the interaction within (left) and between (right) tubulin dimers. **b** Comparison between the frequency for the residues of the central tubulin of forming an interaction with adjacent tubulins, as identified by PLIP, at the B-type and A-type interfaces. Only residues forming an interaction with a frequency greater or equal to 0.75 in at least one system are reported. In this case, the most stable interaction for each residue of the central tubulin was considered, without considering the interaction partner, to compare B-type and A-type contacts

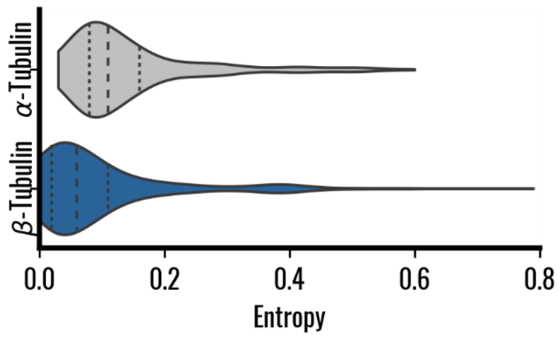

Fig. S4 Evolutionary conservation in Alpha e Beta tubulins. Violinplot showing the distribution of Normalized Entropy in alpha and beta tubulins. The central dashed lines represent the median values, while the lateral dashed lines the 1st and 3rd quartiles

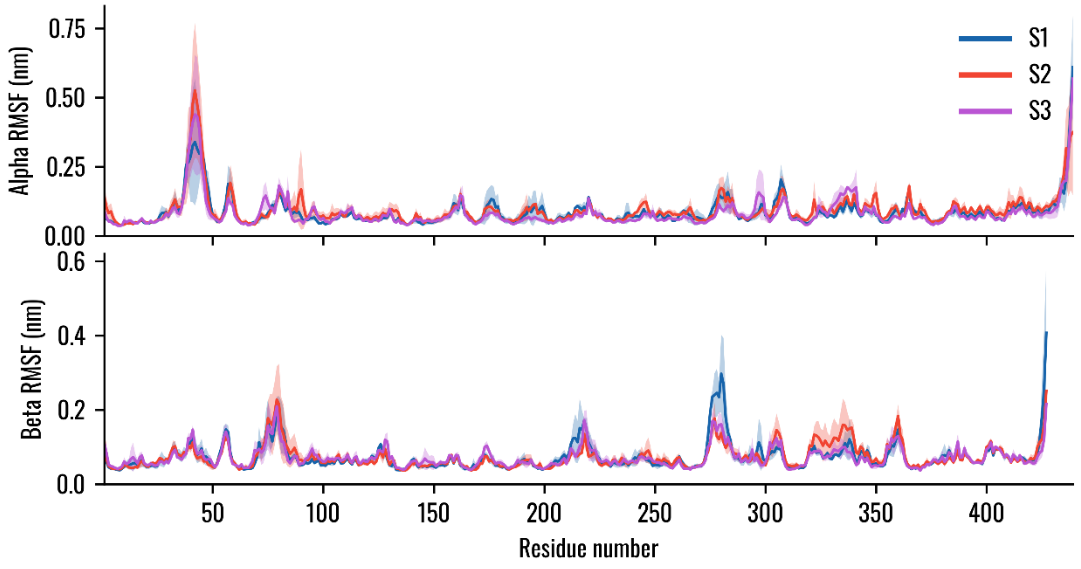

Fig. S5 Residues fluctuations. Root-mean-squared fluctuation of the central tubulin residues (computed on C-alphas). The average value across three systems is represented with a continuous line, while the shaded region highlights the values within one standard deviation from the mean

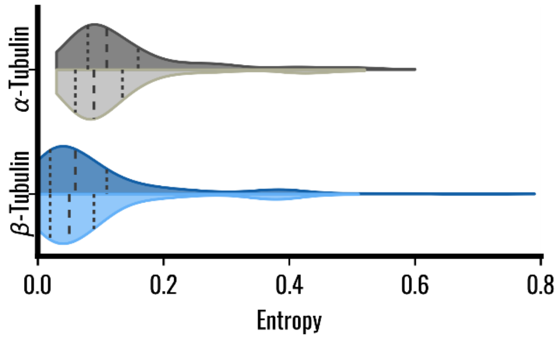

Fig. S6 Conservation of mechanically rigid residues. Violinplot showing the distribution of Normalized Entropy in α and β tubulins, highlighting the difference between the overall distribution (darker color) and the one considering only residues with force constant greater than the median of the profile (lighter color). The central dashed lines represent the median values, while the lateral dashed lines the 1st and 3rd quartiles

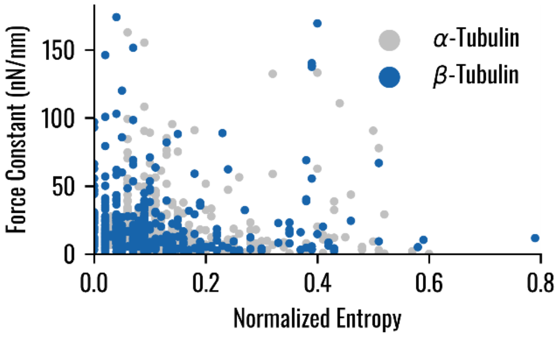

Fig. S7 Scatterplot of normalized entropy and force constants. Residues belonging to α and β tubulins, are coloured in grey and blue respectively

3 References

Adasme MF, Linnemann KL, Bolz SN, et al (2021) PLIP 2021: expanding the scope of the protein–ligand interaction profiler to DNA and RNA. Nucleic Acids Research 49:W530–W534. https://doi.org/10.1093/nar/gkab294

Alushin GM, Lander GC, Kellogg EH, et al (2014) High-Resolution Microtubule Structures Reveal the Structural Transitions in αβ-Tubulin upon GTP Hydrolysis. Cell 157:1117–1129. https://doi.org/10.1016/j.cell.2014.03.053

Berendsen HJC, Postma JPM, Van Gunsteren WF, et al (1984) Molecular dynamics with coupling to an external bath. The Journal of Chemical Physics. https://doi.org/10.1063/1.448118

Cannariato M, Miceli M, Cavaglià M, Deriu MA (2022) Prediction of Protein–Protein Interactions Between Alsin DH/PH and Rac1 and Resulting Protein Dynamics. Front Mol Neurosci 14:772122. https://doi.org/10.3389/fnmol.2021.772122

Gentile F, Deriu M, Licandro G, et al (2015) Structure Based Modeling of Small Molecules Binding to the TLR7 by Atomistic Level Simulations. Molecules 20:8316–8340. https://doi.org/10.3390/molecules20058316

Gowers R, Linke M, Barnoud J, et al (2016) MDAnalysis: A Python Package for the Rapid Analysis of Molecular Dynamics Simulations. Austin, Texas, pp 98–105

Jakalian A, Jack DB, Bayly CI (2002) Fast, efficient generation of high-quality atomic charges. AM1-BCC model: II. Parameterization and validation. J Comput Chem 23:1623–1641. https://doi.org/10.1002/jcc.10128

Lange OF, Grubmüller H (2005) Generalized correlation for biomolecular dynamics. Proteins 62:1053–1061. https://doi.org/10.1002/prot.20784

Lavery R, Sacquin-Mora S (2007) Protein mechanics: a route from structure to function. J Biosci 32:891–898. https://doi.org/10.1007/s12038-007-0089-x

Lindorff-Larsen K, Piana S, Palmo K, et al (2010) Improved side-chain torsion potentials for the Amber ff99SB protein force field. Proteins: Structure, Function, and Bioinformatics 78:1950–1958. https://doi.org/10.1002/prot.22711

Manrique PD, Chakraborty S, Henderson R, et al (2023) Network analysis uncovers the communication structure of SARS-CoV-2 spike protein identifying sites for immunogen design. iScience 26:105855. https://doi.org/10.1016/j.isci.2022.105855

Melo MCR, Bernardi RC, De La Fuente-Nunez C, Luthey-Schulten Z (2020) Generalized correlation-based dynamical network analysis: a new high-performance approach for identifying allosteric communications in molecular dynamics trajectories. The Journal of Chemical Physics 153:134104. https://doi.org/10.1063/5.0018980

Miceli M, Deriu MA, Grasso G (2022) Toward the design and development of peptidomimetic inhibitors of the Ataxin-1 aggregation pathway. Biophysical Journal 121:4679–4688. https://doi.org/10.1016/j.bpj.2022.10.021

Mirny LA, Shakhnovich EI (1999) Universally conserved positions in protein folds: reading evolutionary signals about stability, folding kinetics and function. Journal of Molecular Biology 291:177–196. https://doi.org/10.1006/jmbi.1999.2911

Navizet I, Cailliez F, Lavery R (2004) Probing Protein Mechanics: Residue-Level Properties and Their Use in Defining Domains. Biophysical Journal 87:1426–1435. https://doi.org/10.1529/biophysj.104.042085

Olsson MHM, Søndergaard CR, Rostkowski M, Jensen JH (2011) PROPKA3: Consistent Treatment of Internal and Surface Residues in Empirical pKa Predictions. J Chem Theory Comput 7:525–537. https://doi.org/10.1021/ct100578z

Pallante L, Rocca A, Klejborowska G, et al (2020) In silico Investigations of the Mode of Action of Novel Colchicine Derivatives Targeting β-Tubulin Isotypes: A Search for a Selective and Specific β-III Tubulin Ligand. Front Chem 8:108. https://doi.org/10.3389/fchem.2020.00108

Valdar WSJ (2002) Scoring residue conservation. Proteins 48:227–241. https://doi.org/10.1002/prot.10146

Wang J, Wolf RM, Caldwell JW, et al (2004) Development and testing of a general amber force field. J Comput Chem 25:1157–1174. https://doi.org/10.1002/jcc.20035

(2019) Molecular Operating Environment (MOE)
